# Clear Cell Sarcoma of the Knee: A Case Report of Diagnostic Challenges, Rapid Metastatic Spread, and Treatment Dilemmas

**DOI:** 10.7759/cureus.80996

**Published:** 2025-03-22

**Authors:** Abid Nawaz Khan Adil, Muhammad Hassan, Muhammad Khan, Bakht Rahman, Syed S Raza, Giustino Varrassi

**Affiliations:** 1 Internal Medicine, Community Medical Center, Fresno, USA; 2 Internal Medicine, University of Kansas Medical Center, Kansas City, USA; 3 Physiology, Khyber Medical College/Teaching Hospital, Peshawar, PAK; 4 Pain Medicine, Fondazione Paolo Procacci, Rome, ITA

**Keywords:** clear cell cancer, immune checkpoint inhibitor, knee pain and cancer, ortho oncology, tyrosine kinase inhibitors (tki)

## Abstract

Clear cell sarcoma (CCS) is a rare, aggressive soft tissue sarcoma with a strong predilection for the extremities. It is often misdiagnosed as melanoma. Despite surgical resection, CCS carries a high risk of early metastasis, most commonly to the lungs, bones, and lymph nodes, with limited effective systemic therapies available. We present the case of a 48-year-old man with CCS of the knee, initially presenting as a localized disease but rapidly progressing to widespread metastasis within three months. The patient underwent radical resection with knee arthroplasty and adjuvant radiotherapy, followed by systemic therapy with cabozantinib, ipilimumab, and nivolumab. Despite aggressive treatment, the disease progressed, leading to recurrent pleural effusions, respiratory failure, and, ultimately, the patient's demise within 10 months of diagnosis. This case underscores the highly aggressive nature of CCS and the challenges associated with its management. The rapid metastatic spread despite multimodal therapy highlights the need for improved early detection strategies and more effective systemic treatments, including novel targeted therapies and immunotherapy combinations.

## Introduction

Clear cell sarcoma (CCS) is a rare malignant tumor first described by Enzinger in 1965. It accounts for less than 1% of all sarcomas [1-3]. Historically, CCS was referred to as "melanoma of the soft parts". Upon initial histological examination, it is easily confused with melanoma, although it has distinct cytogenetic characteristics and a different natural history than melanoma [1,4]. Typically, it presents as a localized disease affecting the extremities but can occasionally originate from the trunk or scalp [5]. Early diagnosis and initial radical surgery are crucial for a favorable outcome. Once regional lymph node metastasis or hematogenous dissemination has occurred, the prognosis is dismal [5]. The lung is the most common site of metastasis, followed by bone and distant lymph nodes [6]. Tumor size is the most important prognostic factor, although tumor necrosis has also been found to be a significant indicator of poor prognosis, independent of tumor size [5,7]. We present a case of a 48-year-old man diagnosed with CCS of the knee. Initially appearing as a localized disease, it later metastasized to the lungs and bones in less than three months after the diagnosis. The patient did not respond to multiple lines of treatment and eventually succumbed within 10 months after diagnosis.

## Case presentation

A 48-year-old man with no significant medical history presented to the outpatient department with complaints of left-sided knee swelling and persistent pain for three months. Over the past few weeks, the patient reported the appearance of several hard nodules on the left lateral knee that were tender to palpation. He experienced mild to moderate discomfort in the knee while walking. No focal motor or sensory deficits were observed.

During the general physical examination, hard and mobile masses were palpable over the anterolateral leg. Tenderness was observed upon palpation over the proximal fibula and proximal tibia on the lateral part. The patient had a full active and passive range of motion at the hip, knee, and ankle. No rashes or other unusual lesions were noted on the skin throughout the body.

**Figure 1:**
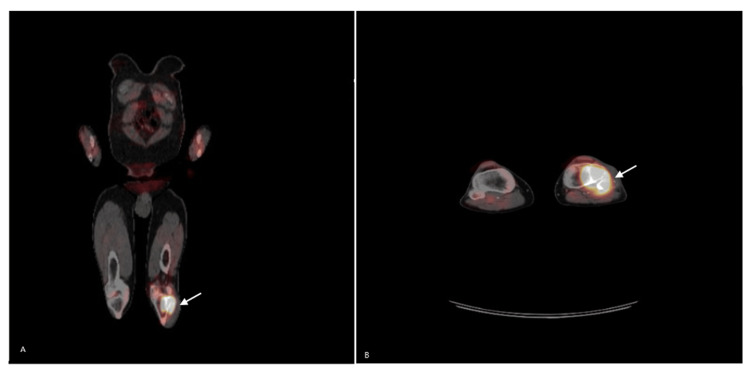
PET scan showing uptake in the left knee A: Osteolytic mass in the left lower knee involving the tibia and fibula with an SUV of 26, indicating high metabolic activity B: A magnified view of the mass in the left lower knee with increased uptake involving the tibia and fibula, along with adjacent soft tissues SUV: standardized uptake value; PET: positron emission tomography

During the initial visit, an ultrasound-guided biopsy was performed on the superficial nodules of the knee. Subsequently, the patient underwent a magnetic resonance imaging (MRI) scan of the left knee, in the following week, revealing a 6 cm mass affecting the lateral part of the tibia, fibula, and anterior compartment. Additionally, a positron emission tomography (PET) scan displayed a large 6.3×4.4×5 cm osteolytic mass involving the medial left tibial plateau and proximal left fibula, along with adjacent soft tissues (Figure 1A, 1B). The scan indicated a high uptake of 26 standardized uptake value (SUV), and no metastatic disease was observed.

Given the presence of a large exophytic mass involving the knee bones and the surrounding soft tissues, differential diagnoses at this point included osteochondroma, giant cell tumor, soft tissue sarcoma, chondrosarcoma, and osteosarcoma. 

**Figure 2:**
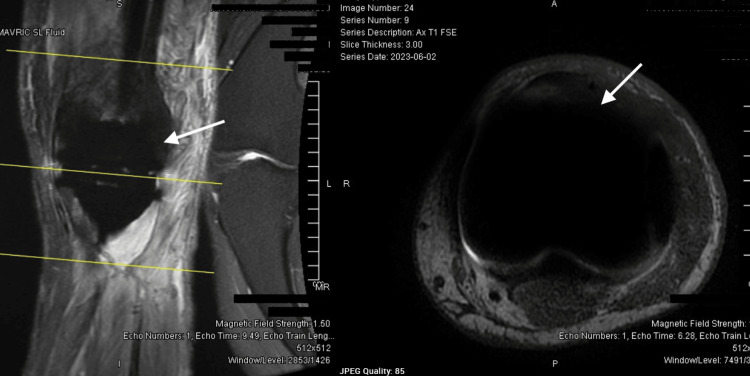
MRI of the left knee post-reconstructive surgery A: Complete excision of the mass from the left knee. Notable post-surgical changes include the appearance of the surgical site and surrounding edema B: Post-reconstructive surgery view of the left knee highlighting the reconstructed structures and the surrounding soft tissues MRI: magnetic resonance imaging

The initial biopsy performed indicated melanoma, despite the absence of visible skin lesions on repeated physical examinations. Subsequently, the report was amended to confirm CCS once gene rearrangement results returned positive for EWSR1-ATF1, consistent with CCS. EWSR1-ATF1 refers to a fusion gene that occurs as a result of a chromosomal translocation. The patient underwent radical resection with reconstructive surgery involving total left knee arthroplasty (Figure 2). Surgical pathology from the procedure also confirmed high-grade CCS with negative margins, although close (<0.1 cm).

The patient underwent laboratory workup at baseline and during treatment. This is presented in Table 1.

**Table 1:** Laboratory investigations at baseline and during treatment WBC: white blood cells; RBC: red blood cells; HGB: hemoglobin; HCT: hematocrit; CO_2_: carbon dioxide; BUN: blood urea nitrogen; BNP: brain natriuretic peptide; LDH: lactate dehydrogenase

	Test	Baseline	During treatment	Reference range
Complete blood count	WBC	15.5×10^3^/µL	14.9×10^3^/µL	4.0-11.0×10^3^/µL
	RBC	3.21×10^6/^µL	3.41×10^6/^µL	4.0-5.0×10^6/^µL
	HGB	9.4 g/dL	9.7 g/dL	12.1-15.1 g/dL
	HCT	28.1%	28.5%	Male: 42-50%
	Platelets	403×10^3^/µL	300×10^3^/µL	150-450×10^3^/µL
Absolute cell count	Neutrophil	12.9	13.1	Cells/microliter
	Lymphocyte	1.4	1.0	Cells/microliter
	Monocyte	1.0	0.7	Cells/microliter
	Eosinophil	0.0	0.0	Cells/microliter
	Basophil	0.0	0.0	Cells/microliter
Comprehensive metabolic panel	Sodium	136 mmol/L	112 mmol/L	135-145 mmol/L
	Potassium	3.9 mmol/L	5.3 mmol/L	3.5-5.0 mmol/L
	Chloride	101 mmol/L	75 mmol/L	95-108 mmol/L
	CO_2_	28 mmol/L	29 mmol/L	20-32 mmol/L
	Glucose	113 mg/dL	125 mg/dL	70-110 mg/dL
	BUN	19 mg/dL	16 mg/dL	8-25 mg/dL
	Osmolality	285 mOsm/kg	237 mOsm/kg	275-295 mOsm/kg
	Creatinine	0.6 mg/dL	0.5 mg/dL	0.6-1.3 mg/dL
	BUN/creatinine	32	32	10:1-20:1
	Albumin	3.1 g/dL	3.3 g/dL	3.5-5.5 g/dL
	Albumin/globulin	0.9	1.1	1.1-2.5
	Alkaline phosphatase	72 IU/L	117 IU/L	30-120 IU/L
	Calcium	8.8 mg/dL	9.2 mg/dL	8.5-10.5 mg/dL
	Iron	Not done	16 μg/dL	50-150 μg/dL
	LDH	150 U/L	254 U/L	60-160 U/L
	Magnesium	2.0 mg/dL	1.7 mg/dL	1.6-2.6 mg/dL
	Transferrin	Not done	185 mg/dL	200-400 mg/dL
	BNP	Not done	470 pg/mL	<100 pg/mL

The patient commenced adjuvant radiotherapy after the decision of the multidisciplinary team (MDT) on the affected left knee one month post-surgery. A three-month follow-up PET scan revealed metastatic disease affecting the bilateral lungs, left inguinal lymph nodes, right clavicular bone, and right shoulder muscles. Due to the extensive metastatic disease identified on the PET scan, radiation therapy was deferred. Instead, the decision was made in the MDT meeting to initiate treatment with cabozantinib at 40 mg daily, along with ipilimumab at 1 mg/kg and nivolumab at 3 mg/kg every three weeks for four doses. Following this, the patient was placed on maintenance nivolumab and cabozantinib. However, cabozantinib was discontinued for two weeks due to the development of hand and foot syndrome. The metastatic disease of the patient is also evident.

**Figure 3:**
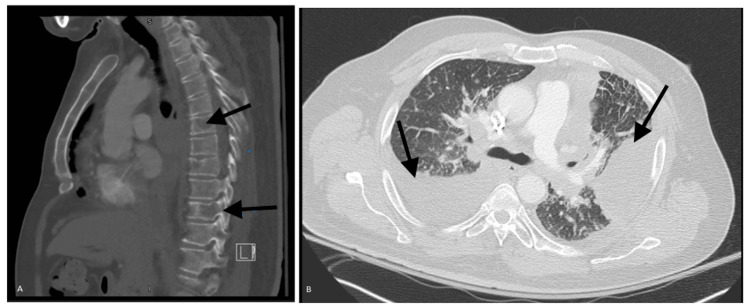
Follow-up CT scan showing disease progression A: Sagittal view of the thoracic spine demonstrating multiple lytic lesions, indicating areas of bone destruction typically seen in metastatic disease or primary bone pathology. The lesions appear as radiolucent areas within the vertebral bodies B: Lung metastasis with associated pleural effusion. The metastatic lesion is identified within the lung parenchyma, and the pleural effusion is noted as a fluid collection in the pleural space, likely secondary to the metastatic spread CT: computed tomography

The patient's condition eventually deteriorated, leading to two hospital admissions due to recurrent large bilateral pleural effusions resulting in hypoxic respiratory failure. Empirical treatment was administered for chemotherapy-induced pneumonitis and atypical pneumonia using steroids and antibiotics, but it didn't improve his symptoms. Despite three thoracenteses, all yielding negative results for malignancy, cancer metastasis remained the primary differential diagnosis for his worsening respiratory status. A computed tomography (CT) scan performed revealed possible metastasis to vertebrae and lung metastasis with pleural effusion (Figure 3A, 3B). Subsequently, a PleurX catheter was placed to provide symptomatic relief from recurrent pleural effusion. The plan was to stabilize the patient before transferring him to a higher level of care for experimental chemotherapy.

Unfortunately, his condition worsened significantly, and he was transferred to palliative care in the hospital, where he passed away.

## Discussion

CCS is a rare melanocytic soft tissue sarcoma, which is easily confused with melanoma due to the presence of immunohistochemical staining for S-100 protein and ultrastructural evidence of melanosomes. However, it is genetically distinct, lacking melanoma-associated BRAF mutations and instead featuring recurrent and characteristic chromosomal translocations involving the EWSR1 gene [4,6]. The majority of cases present as a localized disease affecting the extremities, although it can occasionally originate from the trunk or scalp. The lung is the most common site of metastasis, followed by the bone and distant lymph nodes [5]. In our case, the tumor originated from the knee and later spread to the lungs and distant bones, including the clavicle and vertebral bones.

Tumor size is the most important prognostic factor in CCS. Generally, a tumor size of ≤3 cm is considered a favorable prognostic factor for patient survival in CCS [5,8]. Tumor necrosis is also a significant indicator of poor prognosis, independent of tumor size [5,7]. In our case, the initial tumor size at presentation was 6.3 cm, strongly contributing to the poor prognosis. Early diagnosis and initial radical surgery are crucial for a favorable outcome [1]. Regional lymph node involvement or hematogenous dissemination makes the prognosis poor [9]. If the disease is localized, the survival rates are 59% at five years and 41% at 10 years [1,5]. However, in metastatic settings, the two-year disease-specific survival rates for lymph node and pulmonary metastasis groups are 40% and 0%, respectively [1,5]. Unfortunately, in our case, the patient passed away within 10 months after diagnosis despite undergoing surgery and chemotherapy.

The preferred treatment for localized CCS is wide surgical resection. Despite the optimal management of localized disease, a high proportion of patients develop metastatic disease [10]. Conventional chemotherapy exhibits minimal activity in CCS, as it demonstrates limited sensitivity to anthracycline-based chemotherapy [11]. Tyrosine kinase inhibitors (TKI), including crizotinib, sorafenib, tivantinib, and sunitinib, have been administered in various clinical trials for treating patients with CCS [2]. Generally, responses have been poor, partial, and of very short duration, resulting in limited disease stabilization. Early analyses of novel clinical trials investigating sunitinib or chemotherapy in combination with nivolumab are encouraging [12]. Recently, emerging data from clinical trials evaluating cabozantinib as a treatment for soft tissue sarcoma have shown promising results, with high treatment tolerability and effective disease stabilization [13,14], particularly when cabozantinib is combined with immune checkpoint inhibitors [15]. In our case, the patient was initiated on cabozantinib with ipilimumab and nivolumab early in the disease course; however, the patient did not respond, resulting in a worse outcome. Additionally, the patient experienced a hand-foot skin reaction, which is often reported with the use of cabozantinib [16].

The general treatment of patients such as the one described requires a multimodal approach that also includes the management of collateral symptoms, like pain, which is very disturbing to the quality of life in those patients [17]. A further problem is strictly connected to the side effects of chemotherapy [18]. Recent studies have shown that most of these collateral aspects can be well treated with the use of virtual reality [19]. All these collateral aspects, even if of minor importance compared to the basic pathology, are important to provide the patients with a comfortable and less disturbing therapeutic approach. Hence, they should be always considered as an integral part of the therapy.

## Conclusions

CCS is a rare and aggressive soft tissue sarcoma that predominantly arises in the lower extremities, such as the knee, and is often misdiagnosed as melanoma due to histological similarities. However, CCS has a distinct cytogenetic profile, particularly the EWSR1 gene rearrangement, which differentiates it from melanoma and contributes to its unique clinical course. Despite advances in diagnostic techniques, early detection remains crucial, as radical surgical resection offers the best chance for prolonged survival. However, once regional lymph node metastasis or hematogenous dissemination occurs, the prognosis is poor, with common metastatic sites including the lungs and bones. Conventional chemotherapy has shown limited efficacy, as CCS is generally resistant to anthracycline-based regimens. Recent advances in targeted therapies, particularly TKI such as cabozantinib, in combination with immune checkpoint inhibitors, have demonstrated promising results in stabilizing disease progression in some patients. However, response rates remain variable, highlighting the urgent need for further research and the development of more effective treatment strategies for metastatic CCS. The case presented emphasizes the challenges of managing CCS, especially in the face of rapid metastasis and resistance to current therapeutic approaches, underscoring the necessity of developing novel treatments to improve patient outcomes.
